# Mathematical Model for Estimating the Sound Absorption Coefficient in Grid Network Structures

**DOI:** 10.3390/ma16031124

**Published:** 2023-01-28

**Authors:** Takamasa Satoh, Shuichi Sakamoto, Takunari Isobe, Kenta Iizuka, Kastsuhiko Tasaki

**Affiliations:** 1 FUKOKU Co., Ltd., 6 Showa Chiyoda-machi, Oura-gun, Gunma 370-0723, Japan; 2 Department of Engineering, Niigata University, Ikarashi 2-nocho 8050, Nishi-ku, Niigata 950-2181, Japan; 3 Graduate School of Science and Technology, Niigata University, Ikarashi 2-nocho 8050, Nishi-ku, Niigata 950-2181, Japan

**Keywords:** sound absorption coefficient, porous material, grid network structure, transfer matrix method

## Abstract

Although grid network structures are often not necessarily intended to absorb sound, the gaps between the rods that make up the grid network are expected to have a sound absorption effect. In this study, the one-dimensional transfer matrix method was used to develop a simple mathematical model for accurately estimating the sound absorption coefficient of a grid network structure. The gaps in the grid network structure were approximated as the clearance between two parallel planes, and analysis units were derived to consider the exact geometry of the layers. The characteristic impedance and propagation constant were determined for the approximated gaps and treated as a one-dimensional transfer matrix. The transfer matrix obtained for each layer was used to calculate the sound absorption coefficient. The samples were fabricated from light-curing resin by using a Form2 3D printer from Formlabs. The measurement results showed that a sound absorption coefficient of 0.81 was obtained at the peak when seven layers were stacked. A sensitivity analysis was carried out to investigate the influence of the rod diameter and pitch. The simulated values tended to be close to the experimental values. The above results indicate that the mathematical model used to calculate the sound absorption coefficient is sufficiently accurate to predict the sound absorption coefficient for practical application.

## 1. Introduction

Grid network structures formed by a large number of round or square rods are often used as screens in machine openings and in natural or forced ventilation inlets and outlets. Various experimental studies have investigated the fluid flow characteristics in grid network structures, such as the oscillatory pressure drop when airflow is applied to a mesh screen [1], the use of a wire mesh as a catalyst for soot mitigation [2] and the pressure loss of wire mesh filters [3].

Yamamoto et al. [4] numerically analyzed the acoustic properties of a frame structure, which is similar to a grid network structure. Satoh et al. [5] compared the predicted and experimental results for sound waves incident perpendicular to a group of cylinder axes, including the sound absorption coefficient. In the grid network structure, such sound waves traverse a direction where the size of the gap between the cylinders varies continuously, which is considered to facilitate a high sound absorption coefficient.

In general, thin grid network materials are often treated as acoustically transparent objects. Therefore, they are used in practical applications or experiments to hold acoustic materials. However, if the grid network has a low aperture ratio, an air layer behind it or several stacked layers, its sound absorption may be significant. In a previous study, Iizuka et al. [6] demonstrated the sound absorption effect of laminated wire mesh structures. Dias and Monaragala [7] performed a similar study on the sound absorption of knitted structures and on metamaterials consisting of laminated thin sheets with apertures [8,9,10]. Dias and Monaragala [7] carried out a similar study on the sound absorption of knitted structures.

Although grid network structures are often not necessarily intended to absorb sound, the gaps between the rods that make up the grid network are expected to have a sound absorption effect. Therefore, predicting acoustic properties such as the sound absorption coefficient of a grid network structure is useful in noise engineering because it is a common engineering shape. Being able to predict the sound absorption effect based on the geometric dimensions of the grid network structure and the physical properties of the gas may help in the development of a compact and simple sound absorption mechanism for various applications. It is expected to provide basic knowledge for many applications, such as filters, sifters, and screens. It can also provide insights into the noise control properties of heaters, heat exchangers, catalysts, and other devices. Furthermore, the grid network structure can function as a reflection barrier for electromagnetic waves and as a vibration damper; thus, it can be used as a device that combines these functions with sound reduction.

Although the finite element method [11,12] is useful in the analysis of acoustic metamaterials, the simpler transfer matrix method was used in this work. The one-dimensional transfer matrix method was used to develop a simple mathematical model for accurately estimating the sound absorption coefficient of a grid network structure. The propagation constants and characteristic impedances were derived by using the Navier–Stokes equations and other equations to consider the viscosity of the air. In addition, a simulated analysis was performed to account for the continuous change in the cross-sectional area when the sound waves are incident perpendicular to the rods in a group of cylinder axes [5]. Moreover, a simulated analysis corresponding to the continuous change in the cross-sectional area is attempted by geometrically estimating the surface area of the wall surface and the volume of the void portion in each segmented element correctly and applying it to a two-plane approximation. Finally, the mathematical model was validated by 3D-printing samples and comparing the measured sound absorption coefficients with the predicted values. The measurement results showed that a sound absorption coefficient of 0.81 was obtained at the peak when seven layers were stacked. A sensitivity analysis was performed to evaluate the effects of parameters such as the diameter and pitch on the predicted values.

## 2. Experimental Validation

### 2.1. Measurement Samples

**Figure 1:**
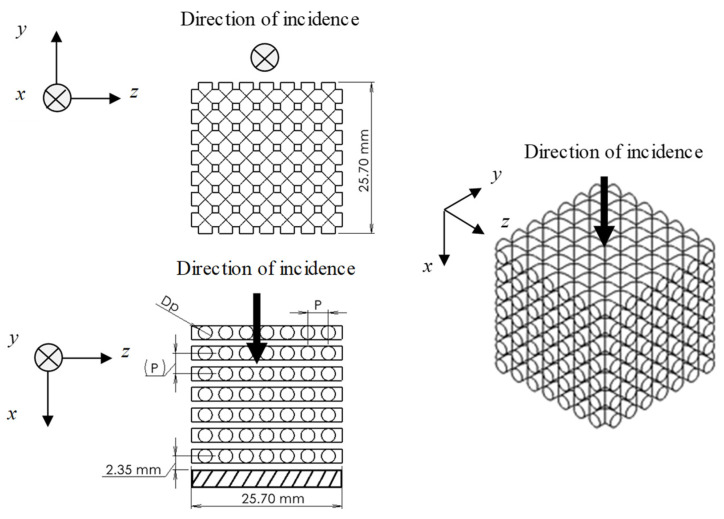
Schematic of a test sample. *D_p_*: rod diameter; *P*: rod pitch; and *N*_m_: number of grids.

**Figure 2:**
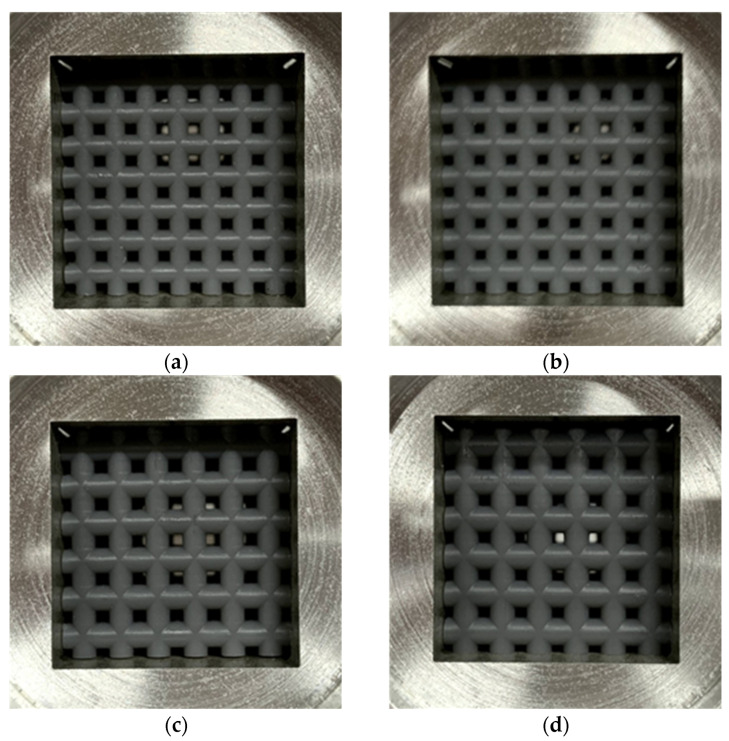
Test samples in sample holder: (**a**) *D_p_* = 2.3 mm, *P* = 3.5 mm, *N*_m_ = 64; (**b**) *D_p_* = 2.5 mm, *P* = 3.5 mm, *N*_m_ = 64; (**c**) *D_p_* = 2.8 mm, *P* = 4.2 mm, *N*_m_ = 49; (**d**) *D_p_* = 3.0 mm, *P* = 4.2 mm, and *N*_m_ = 49.

Figure 1 shows a schematic of the test samples used for the experimental measurements. Figure 2 shows a photograph of the samples. Table 1 presents the sample specifications. The samples were fabricated from acrylic-based light-curing resin by using a Form2 3D printer from Formlabs Inc., (Somerville, MA, USA). The rods comprising the grid network had a length of 25.7 mm, and the diameter and pitch were varied to evaluate their effects on the sound absorption coefficient. Four samples were produced with different dimensions. The 3D printer used a layer pitch of 0.025 mm, which allows a fabrication error of less than 0.05 mm in rod diameter. However, during the initial fabrication runs, the fabrication error of the rod diameters was biased to be several tens of micrometers smaller. To compensate for this, the diameter of the rod on the computer-aided design (CAD) drawing was increased by several tens of micrometers. This resulted in a finished product with more accurate dimensions, as shown in Table 1. The room temperature at the time of fabrication was 20 °C, and the time required to fabricate one sample was approximately 7 h.

The samples were inserted into a sample holder made of aluminum alloy with internal dimensions of 25.7 mm per side for measurement of the sound absorption coefficient. The end faces of the rods and the gap between the bottoms of the sample holder and sample were filled with Vaseline to eliminate factors other than the sample that could contribute to sound absorption.

### 2.2. Measurement Equipment

**Figure 3:**
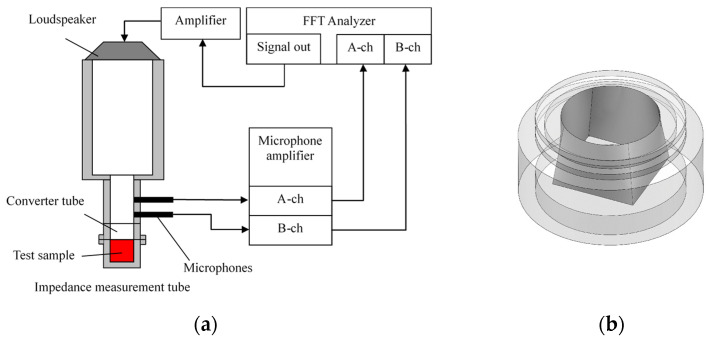
Schema of absorption coefficient measurement: (**a**) two-microphone impedance tube and (**b**) converter tube to convert the cross-sectional shape from circular to square.

A Brüel & Kjær Type 4206 (Brüel & Kjær Sound & Vibration Measurement, Nærum, Denmark)two-microphone acoustic impedance tube system was used to measure the sound absorption coefficient. Figure 3a shows the measurement system. Sound waves with a sinusoidal signal were generated by a signal generator built into the fast Fourier transform (FFT) analyzer and were radiated into the impedance tube by a loudspeaker. The transfer function between the sound pressure signals of two microphones attached to the impedance tube was measured by a FFT analyzer. An Onosokki DS-3000 FFT (Ono Sokki Co., Ltd., Yokohama, Japan) analyzer was used for the measurement. The measured transfer function was then used to calculate the normal incident sound absorption coefficient in accordance with ISO 10534-2 [13]. The derivation of the normal incident sound absorption coefficient is described in detail in ISO 10534-2.

The critical frequency at which a plane wave forms depends on the inner diameter of the impedance tube. In this study, impedance tubes with an inner diameter of 29 mm were used because the sound absorption coefficient is not high at low frequencies. However, a rectangular tube with side lengths of 25.7 mm was used as the sample holder to ensure uniform rod lengths. Thus, a conversion tube was used to smoothly change the cross-sectional shape of the impedance tube from circular to square. Figure 3b shows a perspective view of the conversion tube. The diagonal length of the square cross-section was approximately 36.3 mm, so the limiting frequency of the plane wave was determined as approximately 5400 Hz.

## 3. Simulated Analysis

### 3.1. Transfer Matrix of an Acoustic Element

**Figure 4:**
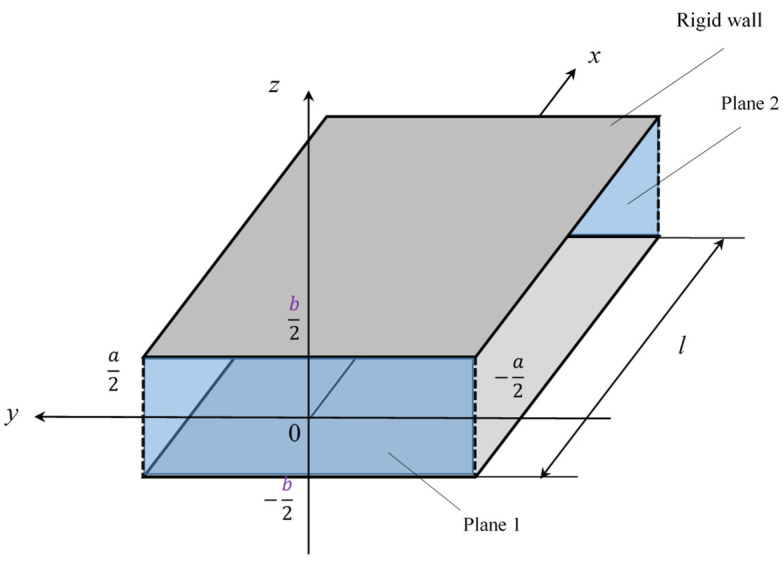
Cartesian coordinate system for the parallel clearance between a pair of planes.

The transfer matrix method was used to calculate the simulated value of the sound absorption coefficient of a grid network structure [14,15]. Figure 4 shows the Cartesian coordinate system for a gap of thickness *b* between two parallel planes 1 and 2. The transfer matrix method can be used to calculate the sound pressure and volume velocity for such a gap based on the one-dimensional wave equation. The relationship between the sound pressure *p*_1_ and volume velocity *Su*_1_ at plane 1 and the sound pressure *p*_2_ and volume velocity *Su*_2_ at plane 2 can be expressed by using the four-terminal constant *t*:

(1) $$\left[\begin{array}{c} p_{1}\\ {Su}_{1} \end{array}\right]=\left[\begin{array}{cc} \cos h\;\gamma l & \frac{Z_{c}}{S}\sin h\;\gamma l\\ \frac{S}{Z_{c}}\sin h\;\gamma l & \cos h\;\gamma l \end{array}\right]\left[\begin{array}{c} p_{2}\\ {Su}_{2} \end{array}\right]=\left[\begin{array}{cc} t_{11} & t_{12}\\ t_{21} & t_{22} \end{array}\right]\left[\begin{array}{c} p_{2}\\ {Su}_{2} \end{array}\right]$$

where *Z_c_* is the characteristic impedance, *γ* is the propagation constant, *S* is the area of the gap and *l* is the length between planes 1 and 2.

### 3.2. Propagation Constant and Characteristic Impedance Considering Attenuation

The propagation constant and characteristic acoustic impedance of small tubes considering attenuation due to air viscosity have been studied for tubes with circular [16,17,18] and equilateral-triangle cross-sections [19].

In this study, Stinson’s method [19] was applied to consider the attenuation of sound waves by the loss of friction due to the viscosity of the boundary layer near the wall. The propagation constant *γ* and characteristic acoustic impedance *Z_c_* of a gap between two surfaces, as shown in Figure 4, can be derived by solving the Navier–Stokes equations, continuity equation, gas state equation, energy equation and dissipative function representing heat transfer. Here, air was assumed to be a compressible fluid, and the air viscosity was assumed to be constant.

The propagation constant *γ* can be expressed as follows:

(2) $$\gamma =\sqrt{\frac{\kappa -\left(\kappa -1\right)B(s\sigma )}{B(s)}},\;B\left(x\right)=1-\frac{\tan hx}{x},\;s=\frac{b}{2}\sqrt{\frac{\rho_{0}\omega }{\mu }}$$

where *κ* is the specific heat ratio, *σ* is the square root of the Prandtl number (0.8677), *μ* is the viscosity (1.869 × 10^−5^ Pa·s), *ω* is the angular frequency and *ρ*_0_ is the density of the gas between two planes. Then, the characteristic impedance *Z_c_* can be expressed as follows:

(3) $$Z_{c}=\frac{p^{+}}{{\bar{u}}^{+}}$$

*u*^+^ and *p*^+^ are the particle velocity and sound pressure, respectively, of the travelling wave:

(4) $${\bar{u}}^{+}=c_{0}B\left(s\right)\frac{j}{\kappa }\left(-\frac{\gamma }{k}\right)\beta e^{-\gamma x}$$

(5) $$p^{+}=P_{s}\beta e^{-\gamma x}$$

where *k* is the wavenumber and *P_s_* is the atmospheric pressure (1.013 × 10^5^ Pa).

### 3.3. Mathematical Model of the Grid Network Structure

The transfer matrix method was used to calculate the sound absorption coefficient of the grid network structure when the sound wave is incident perpendicular to the axial direction of the rods of the grid network structure. Three analysis units were considered to represent different positions in the grid network structure in relation to the tube wall.

#### 3.3.1. Analysis Unit Surrounded by Four Rods

**Figure 5:**
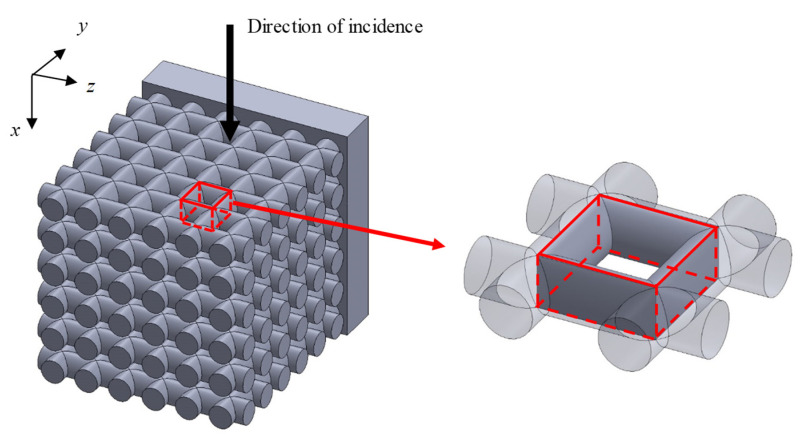
Analysis unit (Unit I) of the grid network structure.

**Figure 6:**
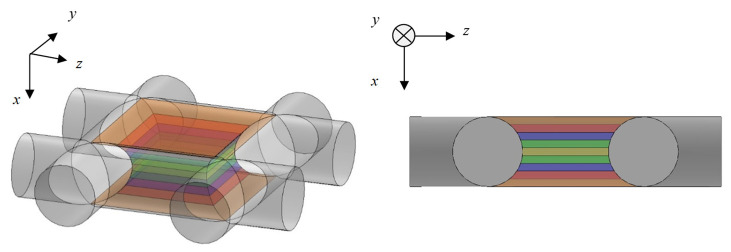
Analysis unit (Unit I) divided into layers of equal thickness in the *x*-axis direction.

**Figure 7:**
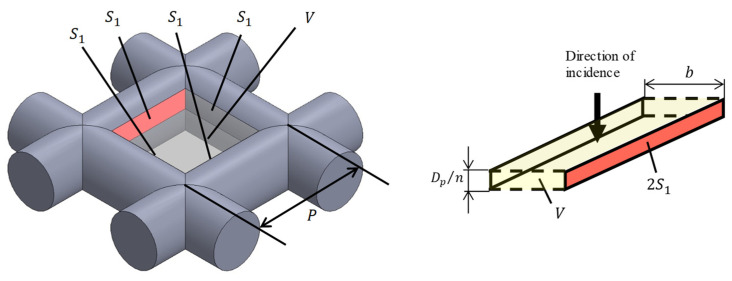
Approximation of analysis unit (Unit I) as the clearance between two planes.

**Figure 8:**
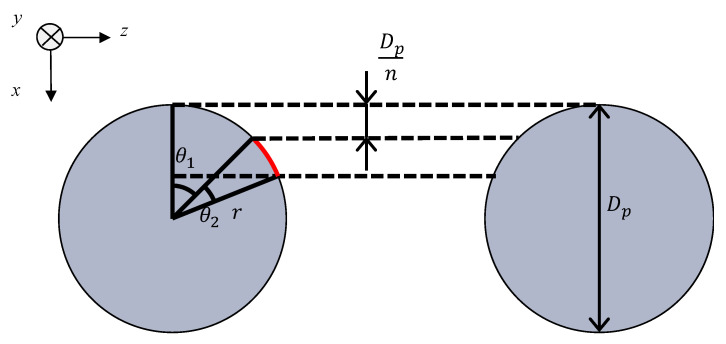
Derivation of surface area *S*_1_.

Figure 5 shows a model of the grid network structure. The first analysis unit (i.e., Unit I) represents an area surrounded by four rods (red rectangle). To obtain the transfer matrix, Unit I is divided into *n* equal layers in the direction of sound incidence (i.e., *x*-axis direction), as shown in Figure 6. The rod diameter is *D_p_*, so the thickness of one layer is *D_p_*/*n*. For the actual analysis, 100 divisions were used because this is when the simulated values converged sufficiently. Then, the clearance between two parallel planes can be approximated by using Allard’s method [20] so that the volume of the void space and the surface area of the rod portion are each equal. Figure 7 approximates a single layer as two parallel planes. The surface area of a wall constituted by one rod is *S*_1_. As shown in Figure 8, the central angle *θ_n_* of the arc constituting *S*_1_ can be expressed as

(6) $$\theta_{n}=\cos^{-1}\left\{1-\left(\frac{x}{r}+\frac{D_{p}}{nr}\right)\right\}-\cos^{-1}\left(1-\frac{x}{r}\right)$$

where *r* is the radius of the rod.

Then, the surface area *S*_1_ is given by

(7) $$S_{1}=\int \left(P-2r\sin\theta \right)rd\theta$$

where *P* is the pitch of the rod. The volume *V* of the void space is given by

(8) $$V=\int {\left(P-2\sqrt{r^{2}-x^{2}}\right)}^{2}dx$$

The thickness in the direction of the sound wave incidence is equal to *D_p_*/*n*, which is the thickness of one layer. The distance *b* between the two planes can be expressed as

(9) $$b=\frac{V}{2S_{1}}$$

where the volume between two parallel planes is *V*. An open-ended correction was added to the grid network structure because the voids can be regarded as orifices in the *x*-axis direction. For the cross-section perpendicular to the direction of sound wave incidence, the orifice was assumed to be in the *x*-axis direction. Then, an open-ended correction length of 0.4 times the radius of a circle equivalent to the area of the smallest gap is applied [21,22]:

(10) $$\Delta l=\frac{P-D_{p}}{\sqrt{\pi }}\times 0.4$$

The open-ended correction length is added to the length *l* of the relevant layer.

#### 3.3.2. Analysis Unit Surrounded by Three Rods and Tube Wall

**Figure 9:**
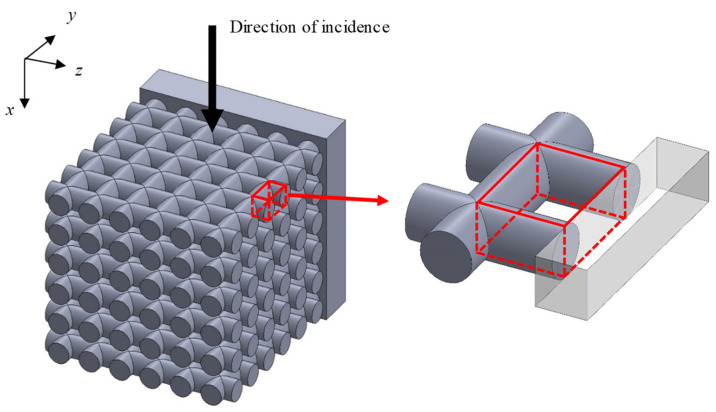
Analysis unit (Unit II) of the grid network structure.

**Figure 10:**
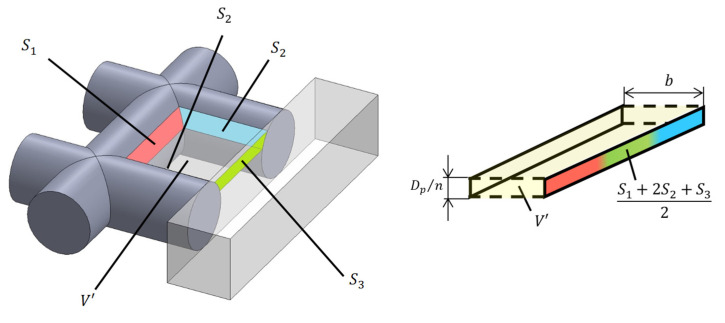
Approximation of Unit II as the clearance between two planes.

Figure 9 shows an analysis unit surrounded by three rods and a tube wall (Unit II). Figure 10 shows the approximation of Unit II as two parallel planes. The lateral surface area *S*_1_ is derived in the same manner as in Unit I. The lateral surface area *S*_2_ is derived in the same manner as in Unit I. The side area *S*_2_ of the rod in contact with the tube wall is derived as follows:

(11) $$S_{2}=\int \left\{\frac{L_{t}-\left(\sqrt{N_{p}}-1\right)P}{2}-r\sin\theta \right\}rd\theta$$

where *L_t_* is the length of one side of the sample holder (i.e., *L_t_* = 25.7 mm).

**Figure 11:**
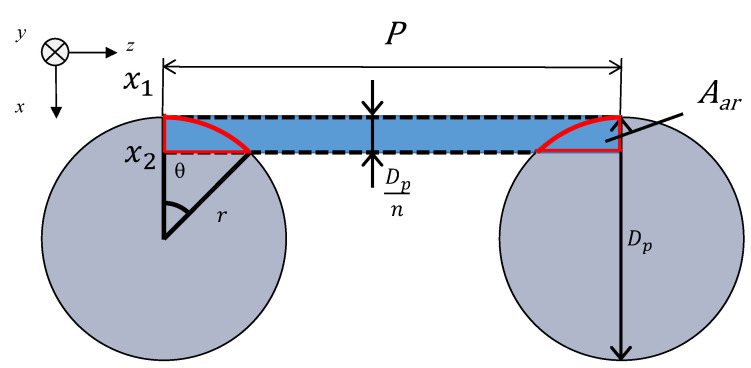
Derivation of surface area *S*_3_.

Figure 11 shows the area *S*_3_ of the inner wall of the sample holder, which is the rectangular area comprising the pitch between the rods and layer thickness minus the area of overlap between the rectangle and end faces of the rods. In other words, *A_ar_* (area enclosed by the red border) can be calculated as follows:

(12) $$A_{ar}=\int_{x1}^{x2}\sqrt{r^{2}-x^{2}}dx$$

Then, the surface area *S*_3_ can be expressed as follows:

(13) $$S_{3}=\frac{D_{p}}{n}\times P-2A_{ar}$$

**Figure 12:**
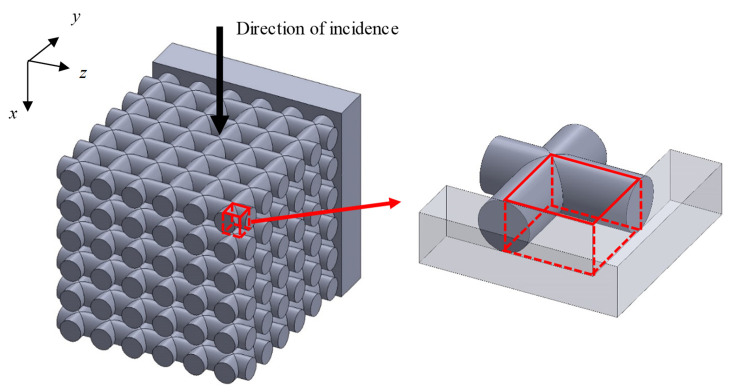
Analysis unit (Unit III) of the grid network structure.

The volume *V*′ in Figure 12 can be expressed as follows:

(14) $$V^{\prime }=\int \left\{\frac{L_{t}-\left(\sqrt{N_{p}}-1\right)P}{2}-\sqrt{r^{2}-x^{2}}\right\}\left(P-2\sqrt{r^{2}-x^{2}}\right)dx$$

Then, the distance *b* between two planes is derived as follows:

(15) $$b=\frac{V^{\prime }}{\frac{S_{1}+2S_{2}+S_{3}}{2}}$$

#### 3.3.3. Analysis Unit Surrounded by Two Rods and Tube Wall

**Figure 13:**
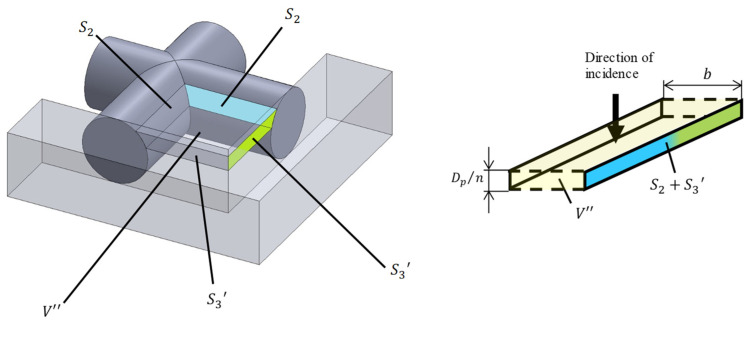
Approximation of Unit III as the clearance between two planes.

Figure 12 shows an analysis unit surrounded by two rods and a tube wall (Unit III). Figure 13 shows the approximation of Unit III as two parallel planes. The lateral area *S*_2_ of the rod tangential to the tube wall is derived in the same way as in Unit II. The area of the tube wall *S*_3_′ is derived as follows:

(16) $${S_{3}}^{\prime }=\frac{D_{p}}{n}\times \frac{L_{t}-\left(\sqrt{N_{p}}-1\right)P}{2}-A_{ar}$$

The volume *V*″ in Figure 13 can be expressed as follows:

(17) $$V^{\prime \prime }=\int {\left\{\frac{L_{t}-\left(\sqrt{N_{p}}-1\right)P}{2}-\sqrt{r^{2}-x^{2}}\right\}}^{2}dx$$

Then, the distance *b* between the two planes can be derived as follows:

(18) $$b=\frac{V^{\prime \prime }}{S_{2}+{S_{3}}^{\prime }}$$

### 3.4. Transfer Matrix of the Grid Network Structure

#### 3.4.1. Transfer Matrix of Analysis Units

**Figure 14:**
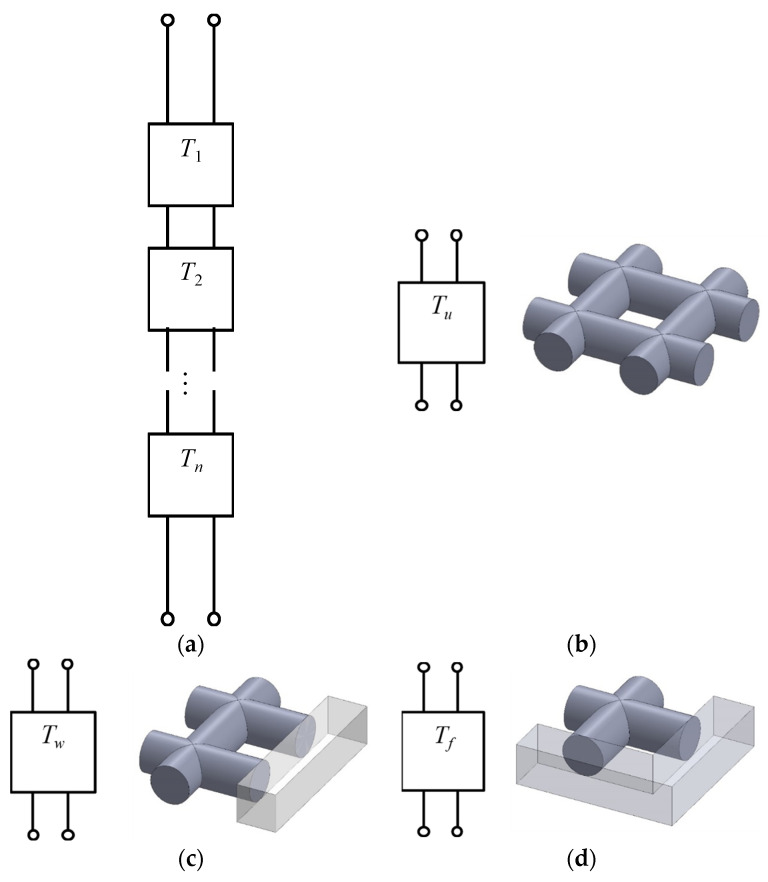
Equivalent circuit analysis: (**a**) cascade connecting *T_n_*, (**b**) summation of all *T_n_* for Unit I, (**c**) summation of all *T_n_* for Unit II, and (**d**) summation of all *T_n_* for Unit III.

The two parallel planes of each layer in an analysis unit correspond to the planes in Figure 4. Thus, Equations (2) and (3) can be used to determine the propagation constant and characteristic impedance for each layer. The propagation constant and characteristic impedance can then be substituted into Equation (1) to obtain the transfer matrices *T*_1_, *T*_2_, *T*_3_, …, *T_n_* for the layers, where the subscript 1 represents the layer at the plane incident to the sound wave, and the subscript *n* represents the number of layers. These transfer matrices can then be cascaded, as shown in Figure 14a, to obtain the transfer matrix for an analysis unit.

Here, *T_u_* is the transfer matrix of Unit I, *T_w_* is the transfer matrix of Unit II and *T_f_* is the transfer matrix of Unit III.

#### 3.4.2. Transmission Matrix of the Whole Sample

**Figure 15:**
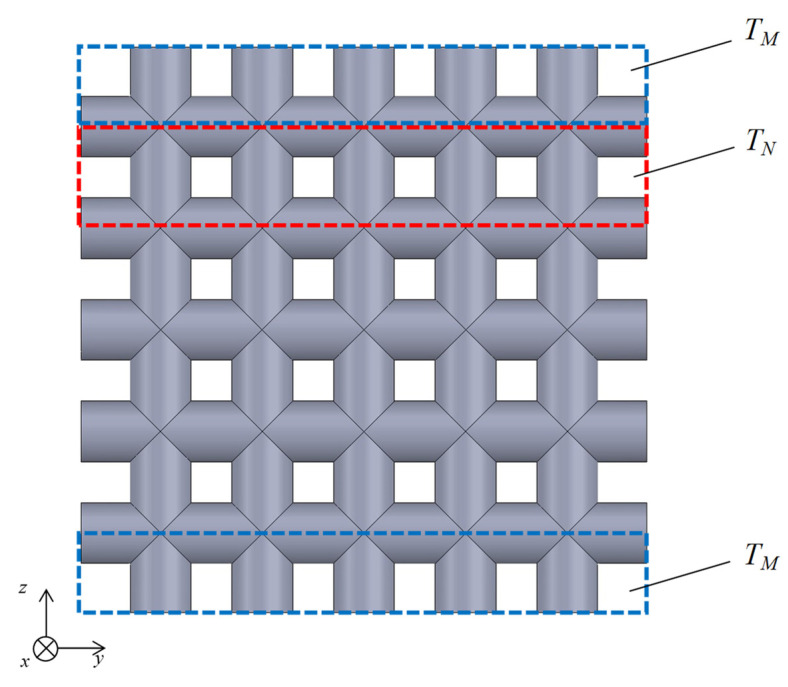
Parallel connection of transfer matrices in a grid network structure.

Figure 15 shows that the transfer matrices of each analysis unit are connected in parallel along the *y*-axis and then along the *z*-axis. This allows the transfer matrix *T_l_* per grid network layer to be derived.

**Figure 16:**
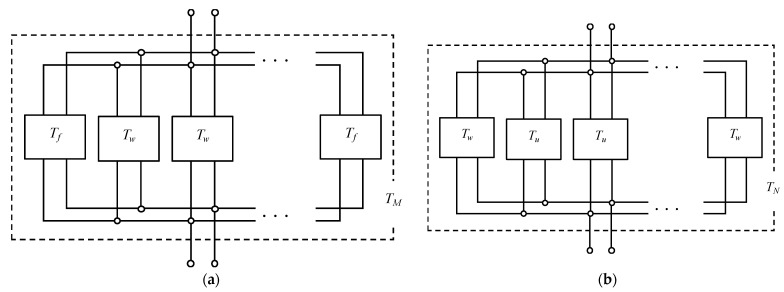
Equivalent circuit of parallel connections in the *y*-axis direction: (**a**) *T*_*w*_ and *T*_*f*_ and (**b**) *T*_*u*_ and *T*_*w*_.

**Figure 17:**
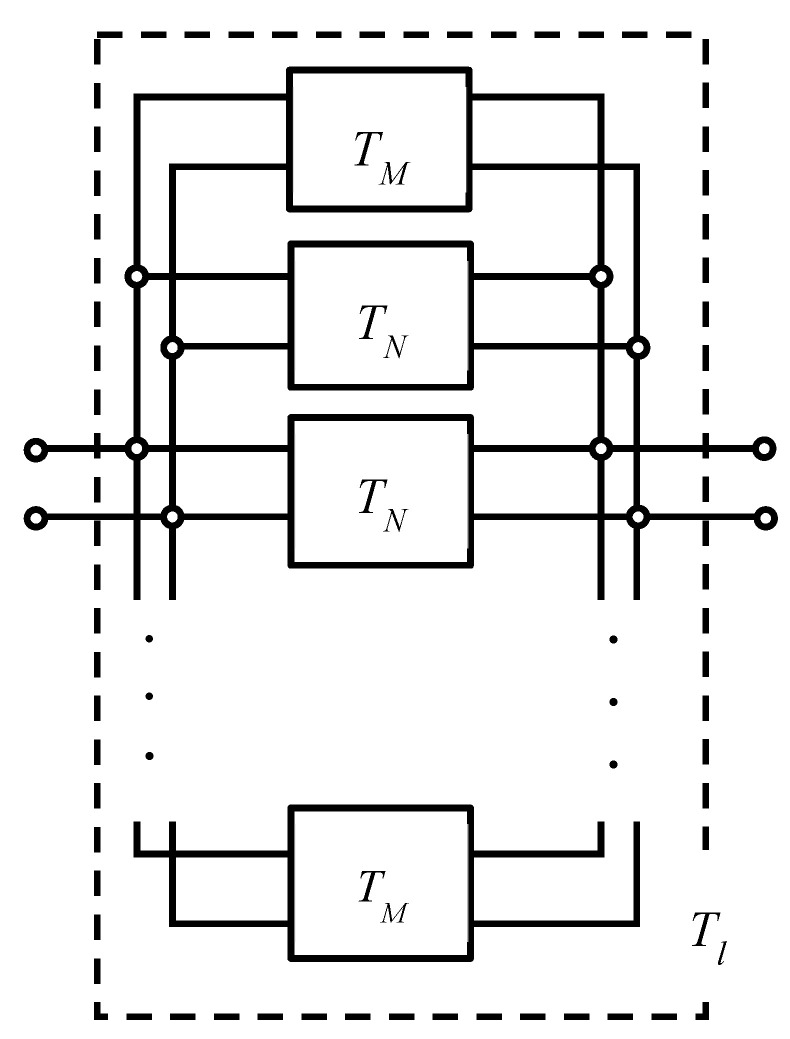
Equivalent circuit of the whole sample as parallel connections of *T*_*M*_ and *T*_*N*_ in the *z*-axis direction.

As shown in Figure 15 and Figure 16, the transfer matrices *T_M_* and *T_N_* are calculated for each analysis unit in parallel in the *y*-axis direction and for one row in the *y*-axis direction, respectively. The number of parallel connections of *T_w_* in *T_M_* and *T_u_* in *T_N_* is one less than the number of rods in the *y*-axis direction. As shown in Figure 17, the calculated *T_M_* and *T_N_* are connected in parallel in the *z*-axis direction. Then, the transfer matrix *T_l_* of the entire sample can be calculated. In this case, the number of *T_N_* in parallel is one less than the number of rods in the *z*-axis direction.

**Figure 18:**
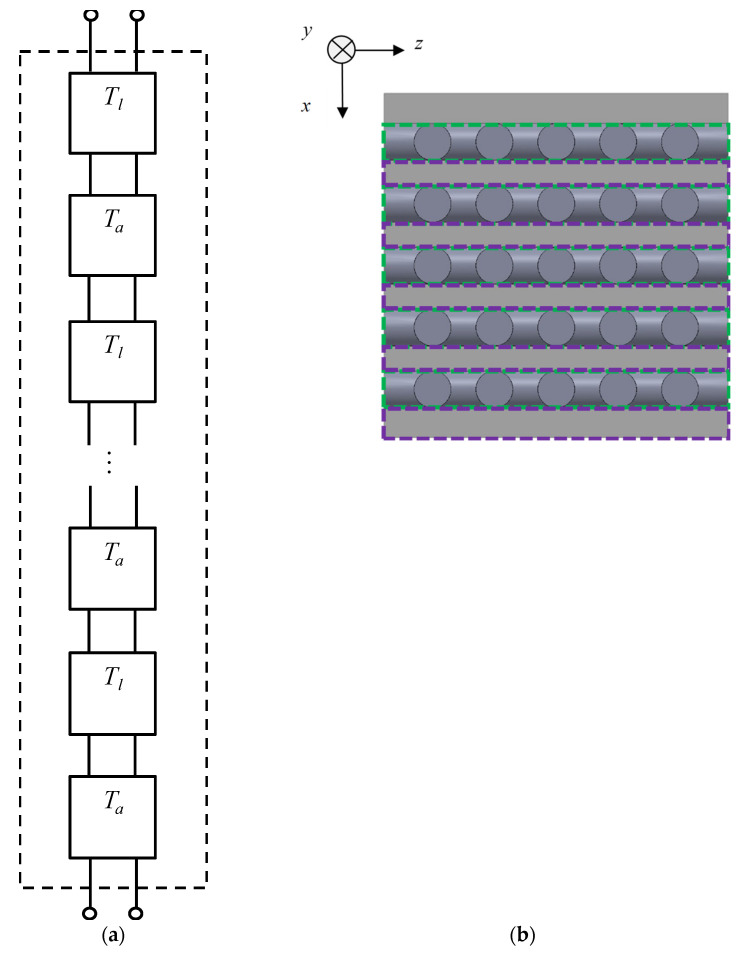
Equivalent circuit of analysis unit: (**a**) cascade connecting *T_l_*, *T_a_* and *T_all_*; and (**b**) cross-section of the grid network structure.

Next, the transfer matrix *T_all_* for the entire sample is derived. As shown in Figure 18, the transfer matrix *T_l_* for one grid network layer and the transfer matrix *T_a_* corresponding to the air layer are connected in an alternating cascade. This allows the entire transfer matrix to be derived.

### 3.5. Derivation of the Sound Absorption Coefficient

The transfer matrix can be used to derive the sound absorption coefficient. The end of the gap was assumed to be a rigid wall, so the particle velocity *u*_2_ = 0. Then, Equation (1) can be transformed as follows:

(19) $$\left[\begin{array}{c} p_{1}\\ {Su}_{1} \end{array}\right]=\left[\begin{array}{cc} t_{11} & t_{12}\\ t_{21} & t_{22} \end{array}\right]\left[\begin{array}{c} p_{2}\\ 0 \end{array}\right]=\left[\begin{array}{c} t_{11}p_{2}\\ t_{21}p_{2} \end{array}\right]$$

As shown in Figure 4, *p*_0_ and *u*_0_ are the sound pressure and particle velocity, respectively, just outside plane 1. If *p*_0_ = *p*_1_ and *S*_0_*u*_0_ = *Su*_1_, then Equation (19) can be used to obtain the specific acoustic impedance *Z*_0_ looking inside the sample from the plane of incidence:

(20) $$Z_{0}=\frac{p_{0}}{u_{0}}=\frac{p_{0}}{u_{0}S_{0}}S_{0}=\frac{p_{1}}{u_{1}S}S_{0}=\frac{t_{11}}{t_{21}}S_{0}$$

The relationship between the specific acoustic impedance *Z*_0_ and reflectance *R* is expressed as follows:

(21) $$R=\frac{Z_{0}\;{-\;\rho }_{0}c_{0}}{Z_{0}{+\rho }_{0}c_{0}}$$

Equation (21) can then be used to obtain the sound absorption coefficient *α*:

(22) $${\alpha =1\;-\;|R|}^{2}$$

## 4. Results

### 4.1. Experimental and Simulated Values of Sound Absorption Coefficient

**Figure 19:**
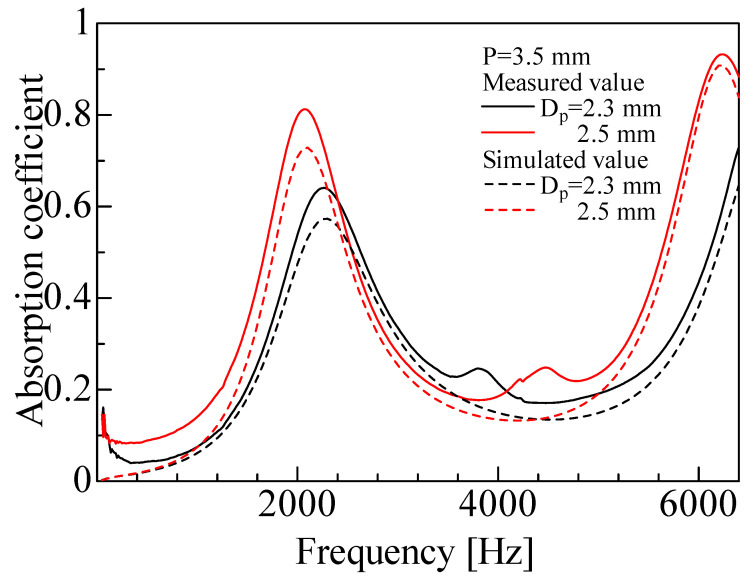
Comparison between experimental and simulated values (*D_p_* = 2.3, 2.5 mm, *P* = 3.5 mm). The rod diameters used in the calculations are the measured values given in Table 1.

**Figure 20:**
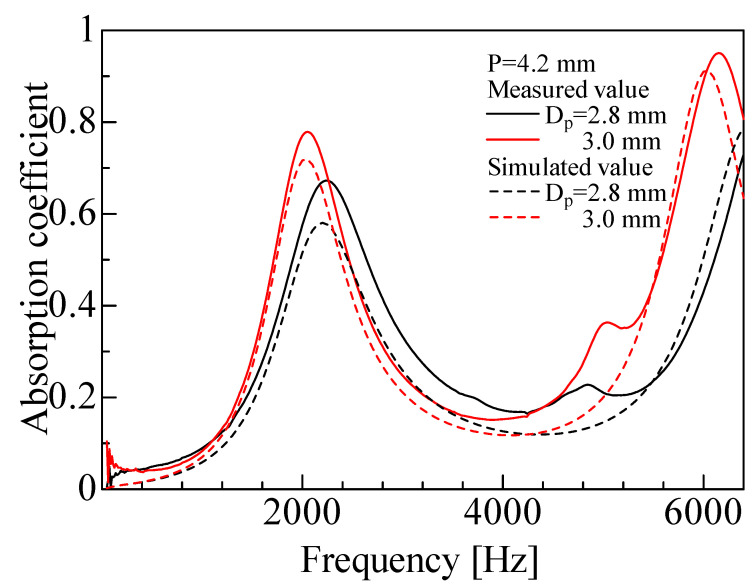
Comparison between experimental and simulated values (*D_p_* = 2.8, 3.0 mm, *P* = 4.2 mm). The rod diameters used in the calculations are the measured values given in Table 1.

The experimental and simulated sound absorption coefficients were compared for each sample. Figure 19 shows the experimental and simulated values for the rod diameters *D_p_* = 2.3 and 2.5 mm and pitch *P* = 3.5 mm. Figure 20 shows the results for the rod diameters *D_p_* = 2.8 and 3.0 mm and pitch *P* = 4.2 mm.

For all samples, the experimental and simulated values generally showed similar trends. The simulated values showed high prediction accuracy, but tended to be lower than the experimental values at peak frequencies. A similar tendency was previously observed by Sakamoto et al. [23] when the one-dimensional transfer matrix method was used to estimate the sound absorption coefficient for other cross-sectional shapes. The difference between the simulated and experimental values may be attributed to the simulated calculation underestimating the sound wave attenuation. The reason for this can be that the particle velocity distribution of the sound wave between the two surfaces was averaged in this simulated analysis.

When sound waves are incident to a gap, the distribution of the *x*-axial component of the particle velocity in the *z*-axis direction within the gap generally has a distribution with respect to the distance from the wall. In contrast, the present simulated analysis assumed the *x*-axial component of the particle velocity in the gap between two planes to have a uniform distribution in the *z*-axis direction. This assumption may have led to the difference between the experimental and simulated values.

### 4.2. Sensitivity Analysis

In this section, simulation results are presented for different wire diameters and pitches.

**Figure 21:**
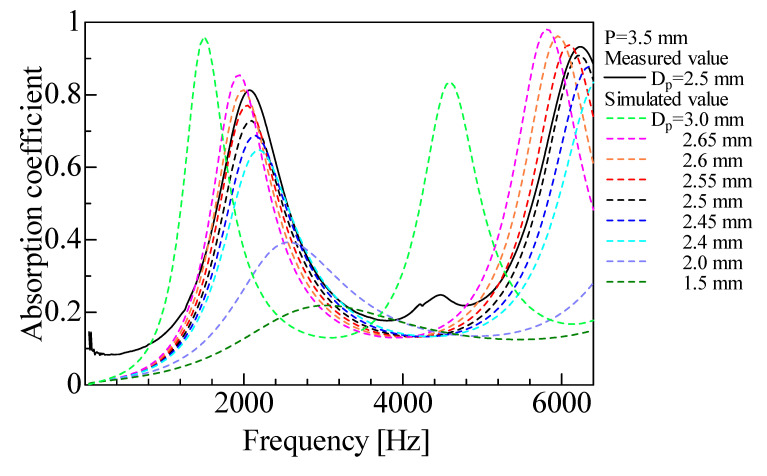
Comparison between experimental and simulated values with changing rod diameter (*D_p_* ≂ 1.5–3.0 mm, *P* = 3.5 mm).

For the sensitivity analysis, the sample shown in Figure 2b (i.e., *D_p_* = 2.5 mm, pitch *P* = 3.5 mm, number of grid network layers *N*_1_ = 7) was used as the baseline. Figure 21 shows the calculated results when the rod diameter *D_p_* was varied. The corresponding experimental result is shown for reference. When *D_p_* was incremented 0.1 mm larger than the actual value (i.e., *D_p_* = 2.6 mm), the simulated and experimental values were almost identical. Therefore, the difference between the simulated and experimental values can be attributed to a difference in the rod diameter of approximately 0.1 mm. One way to reduce the difference between the experimental and simulated values to an acceptable level is to adjust the rod diameter used in the simulated calculation.

Figure 21 also shows that the peak value of the sound absorption coefficient increased with an increasing rod diameter, and the peak frequency tended to shift lower. This may be attributed to the increasing influence of the boundary layer as the gap becomes smaller. In general, the peak becomes sharper as the aperture ratio decreases. Here, the sound absorption peak also became sharper as the rod diameter increased (i.e., as the aperture ratio decreased).

**Figure 22:**
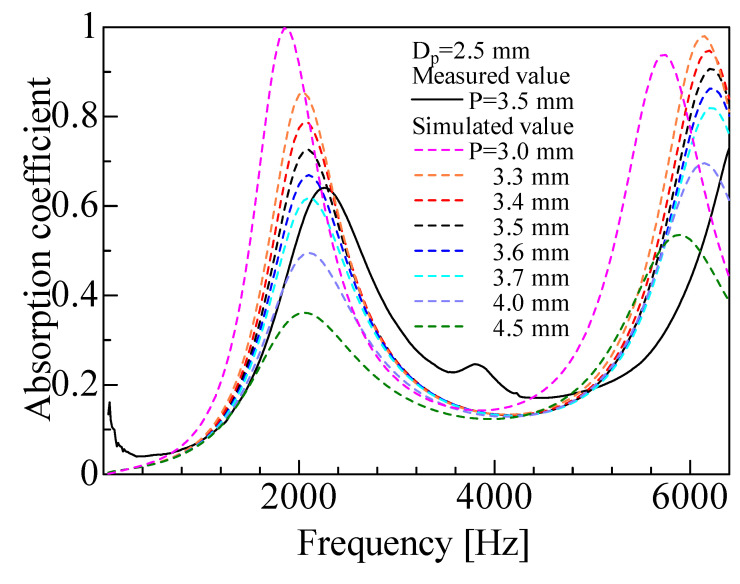
Comparison between experimental and simulated values with changing pitch (*P* ≂ 3.3–3.7 mm, *D_p_* = 2.5 mm).

Figure 22 shows the calculated results when the pitch *P* was varied. The size of the sample tube was changed to match the change in pitch. A smaller pitch tended to increase the peak value of the sound absorption coefficient. This may be because a smaller pitch decreases the gap, which again increases the influence of the boundary layer.

**Figure 23:**
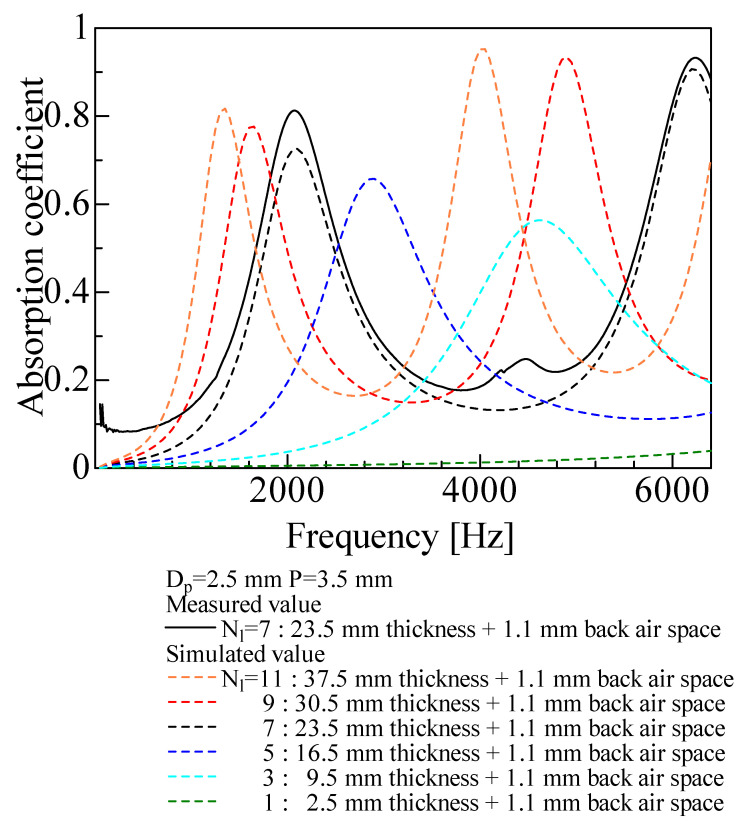
Comparison between experimental and simulated values with changing number of layers (*N_l_* ≂ 1–9).

Figure 23 shows the calculated results when the number of grid network layers *N*_l_ was varied. The peak value increased with the number of layers, and the peak frequency tended to shift lower. This may be because an increase in the number of grid network layers corresponds to an increase in the thickness of the sound-absorbing material. For a single grid network layer, the sound absorption coefficient was difficult to confirm.

**Figure 24:**
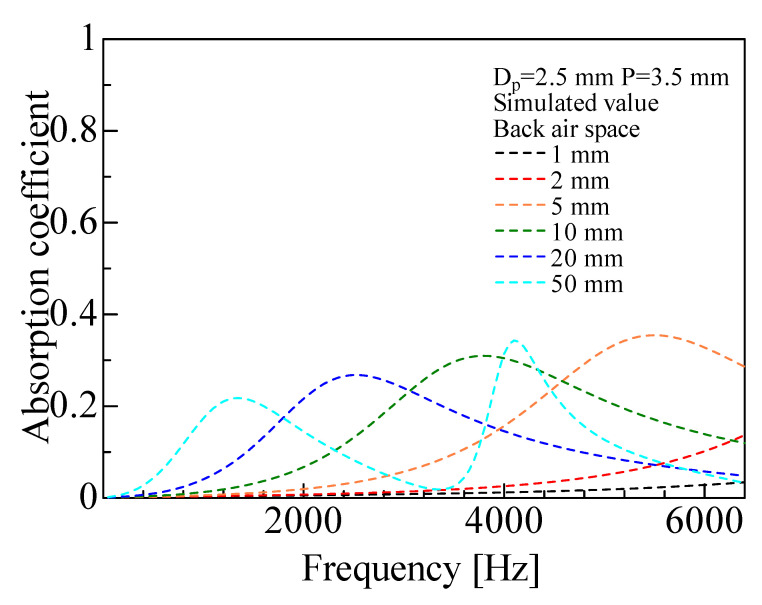
Simulated values with a changing length of back air space (1–9 mm) (*N_l_* = 1, *D_p_* = 2.5 mm, *P* = 3.5 mm).

Figure 24 shows the calculation results for one grid network layer when the thickness of the back air layer was varied. Increasing the thickness of the back air layer decreased the peak value of the sound absorption coefficient, and the peak frequency tended to shift lower. This confirmed that a sound absorption effect could be achieved by the back air layer even when there was only one grid network layer.

These results show that the parameters used in the simulated analysis can be varied to explore their effects on the sound absorption coefficient. The comparison between the experimental and simulated results show that the observed trends for the changes in the sound absorption coefficient are reasonable.

## 5. Conclusions

A mathematical model of a grid network structure consisting of rods was developed to estimate the sound absorption coefficient, and the simulated results were compared with experimental measurements. The gaps in the grid network structure were approximated as the clearance between two parallel planes, and analysis units were derived to consider the exact geometry of the layers. The characteristic impedance and propagation constant were determined for the approximated gaps and treated as a one-dimensional transfer matrix. The transfer matrix obtained for each layer was used to calculate the sound absorption coefficient.

The simulated values tended to be close to the experimental values, but generally underestimated them. This may be because the simulated analysis underestimated the sound wave attenuation. For the peak frequency, the simulated analysis showed high prediction accuracy for the peak frequency. The measurement results show that the rod diameter of 2.5 mm, pitch of 3.5 mm, and the presence of seven layers resulted in a sound absorption coefficient of 0.81 at the first peak.

A sensitivity analysis was carried out to investigate the influence of the rod diameter and pitch. The peak sound absorption coefficient tended to increase with an increasing rod diameter and decreasing pitch. This was attributed to the increasing influence of the boundary layer as the gaps become smaller. The above results indicate that the mathematical model used to calculate the sound absorption coefficient is sufficiently accurate to predict the sound absorption coefficient for practical applications. Thus, it was found that the difference between the simulated and experimental values was approximately equivalent to 0.1 mm in diameter. Therefore, it is possible to reduce the difference between the experimental and calculated values to a practically acceptable level by adjusting the diameter of the rod.

## Figures and Tables

**Table 1:** Specifications of test samples.

Diameter of Rods [mm] (Measured)	Pitch of Rods [mm]	Height of Test Sample [mm]	Number of Grids *N_m_*	Number of Layers *N_l_*	Correspondence to Figure
2.3 (2.31)	3.5	25.7	64	7	Figure 2a
2.5 (2.51)					Figure 2b
2.8 (2.80)	4.2		49	6	Figure 2c
3.0 (3.02)					Figure 2d

## Data Availability

Data is contained within the article.
